# Supplementary figures and images for: Genome-Wide Identification of the TIFY Family in Longan and Their Potential Functional Analysis in Anthocyanin Synthesis
Source: Biology (Basel). 2025 Apr 1;14(4):364. doi: 10.3390/biology14040364 (PMC12024962; doi:10.3390/biology14040364)

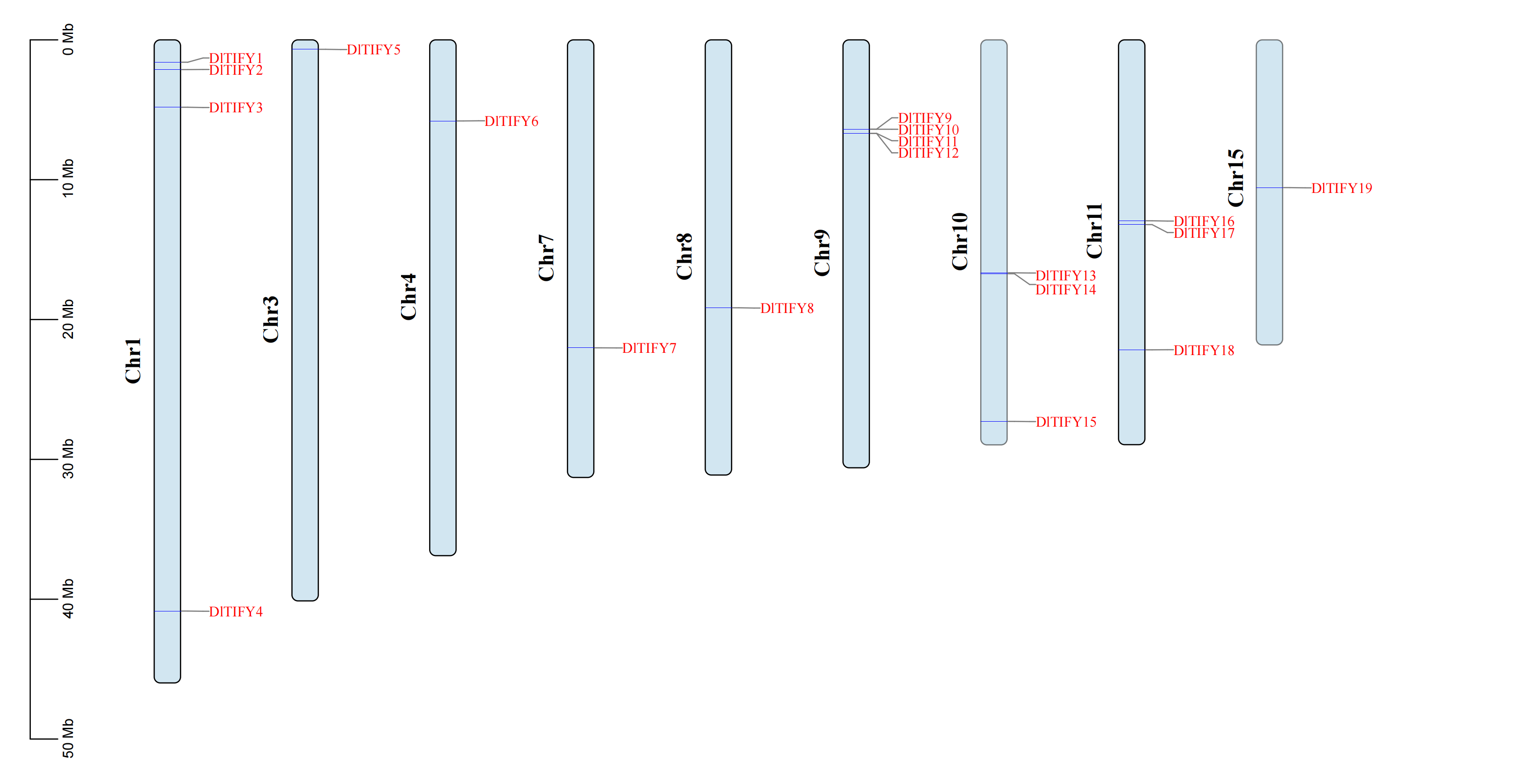

Supplement: Supplementary file 1 [file biology-14-00364-s001.zip › Supplementary File/Figure S1.png]
